# Genes related to the very early stage of ConA-induced fulminant hepatitis: a gene-chip-based study in a mouse model

**DOI:** 10.1186/1471-2164-11-240

**Published:** 2010-04-15

**Authors:** Feng Chen, Hai-Hong Zhu, Lin-Fu Zhou, Jie Li, Li-Ying Zhao, Shan-Shan Wu, Jing Wang, Wei Liu, Zhi Chen

**Affiliations:** 1 State Key Laboratory of Infectious Disease Diagnosis and Treatment, First Affiliated Hospital, College of Medicine, Zhejiang University, Zhejiang, 310003, China; 2 Department of Biochemistry, College of Medicine, Zhejiang University, Zhejiang, 310003, China

## Abstract

**Background:**

Due to the high morbidity and mortality of fulminant hepatitis, early diagnosis followed by early effective treatment is the key for prognosis improvement. So far, little is known about the gene expression changes in the early stage of this serious illness. Identification of the genes related to the very early stage of fulminant hepatitis development may provide precise clues for early diagnosis.

**Results:**

Balb/C mice were used for ConA injection to induce fulminant hepatitis that was confirmed by pathological and biochemical examination. After a gene chip-based screening, the data of gene expression in the liver, was further dissected by ANOVA analysis, gene expression profiles, gene network construction and real-time RT-PCR.

At the very early stage of ConA-triggered fulminant hepatitis, totally 1,473 genes with different expression variations were identified. Among these, 26 genes were finally selected for further investigation. The data from gene network analysis demonstrate that two genes, MPDZ and Acsl1, localized in the core of the network.

**Conclusions:**

At the early stages of fulminant hepatitis, expression of twenty-six genes involved in protein transport, transcription regulation and cell metabolism altered significantly. These genes form a network and have shown strong correlation with fulminant hepatitis development. Our study provides several potential targets for the early diagnosis of fulminant hepatitis.

## Background

Fulminant hepatitis mainly develops from chronic or acute hepatitis B virus (HBV) infection. It has been reported that world widely about 400 million people are living with chronic HBV infection. While 500,000 people die from HBV-related liver cirrhosis and hepatocellular carcinoma, acute fulminant hepatitis B causes additional 40,000 deaths each year[1].

Despite the significant improvement in the antiviral treatment targeting HBV infection, the morbidity and mortality of fulminant hepatitis keep at a high level. This is at least partly due to our poor understanding of the pathogenesis of the disease and the lack of early diagnosing/warning system which lead to a delayed medical intervention. As a serious illness, fulminant hepatitis is not only a threat to our health, but a big financial burden to the patient's family. Therefore, an accurate diagnosis at an early stage is linked directly to an effective treatment, better recovery and lowered mortality of the disease.

Because it resembles very much the human fulminant hepatitis, concanavalin A (ConA)-induced hepatitis has long been used as an experimental model of immune-mediated liver disease [2]. It is characterized by massive hepatocellular degeneration and lymphoid infiltration of the liver [3]. The development of ConA-induced hepatitis depends mainly upon T cell activation [4] which raises plasma levels of various cytokines including TNF-α, interferon (IFN)-γ, and interleukin (IL)-6 [5]. To investigate the pathogenic mechanism of human fulminant hepatitis, in this study, we utilized the ConA-induced hepatitis model and selected microarray as our primary detection tool.

Recently, high-throughput gene chip has increasingly been used to explore the transcription dynamics on a genome-wide scale. This raises a possibility that similar approach could be used to identify gene expression at a specific condition such as the very early stage of fulminant hepatitis. Then the alternations in gene expression could possibly be used as diagnostic measures. Given this aim, it is necessary to use statistical methods to help identify genes whose transcript profiles respond to liver damage. In addition, such approach involves modeling the association of a generic response with a specific experimental variable, such as timing, tissues impacted, temperature, or drug dosage. Therefore, whole genome expression analysis provides a system-level insight for understanding crucial factors that control the liver damage and in turn may help us elucidate the molecular mechanisms of liver injury.

In this study, by using the ConA-induced mouse fulminant hepatitis model, we analyzed the hepatic gene expression with whole genome mouse GeneChip (Affymetrix). Through an integrative analysis that combines changes in gene expression with gene function within a genetic network, we identified 26 genes that may be related to ConA-induced liver injury.

## Results

### ConA-injection induces fulminant hepatitis in Balb/C mice

**Figure 1:**
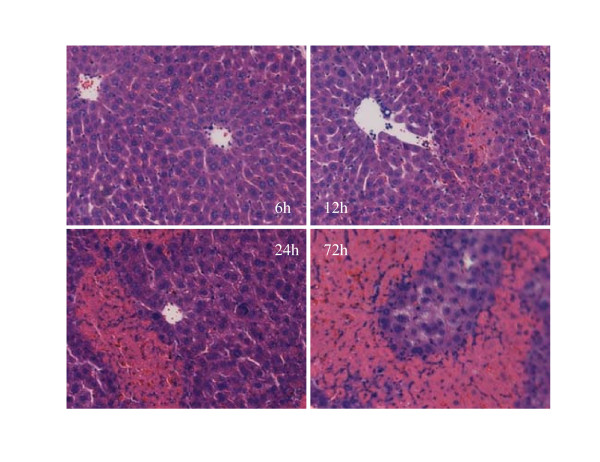
**Pathological examination of liver in mice model with fulminant hepatitis induced by ConA-injection**. ConA induced fulminant hepatitis was confirmed by using pathological examination. Mice liver tissue was obtained at indicated time periods and stained with haematoxylin and eosin. ConA caused pathological changes in mice liver after ConA challenge from the photomicrographs presented. Necrosis appeared at 12 h after ConA administration and the severity of necrosis increased with the time. A large area of necrosis was found at 72 h after ConA injection.

Pathological examination at indicated time periods after ConA challenge showed that fulminant hepatitis mouse model was successfully made. As shown in Figure 1, a single injection of ConA led to a clear liver damage as presented by large area of necrosis (Figure 1). In order to study the changes of very early stage of fulminant hepatitis, liver specimen were taken from the mice at 1 h, 3 h, and 6 h after ConA administration for the further investigation.

### Biochemical evidence for ConA-induced hepatitis

To observe the liver damage in our ConA-induced hepatitis model, we measured by using the classical Reitman-Frankel method the serum levels of alanine aminotransferase (ALT), aspartate aminotransferase(AST), serum total bilirubin (STB) and serum lactate dehydrogenase (LDH). See details in Table 1.

**Table 1:** The ALT, AST, STB and LDH levels in mouse serum after ConA administration

	ALT	AST	STB	LDH
Control group	38.33 ± 1.53	85.33 ± 4.04	2 ± 0	832.67 ± 94.00

1 h	64.67 ± 7.51Δ	194.67 ± 60.90Δ	3.67 ± 0.58Δ	980.0 ± 197.38

3 h	66.67 ± 12.50Δ	222.38 ± 113.40	3 ± 1.00	2454.78 ± 198.62

6 h	1068.17 ± 418.08	768.0 ± 729.61Δ	4.83 ± 0.60Δ	3514.33 ± 977.57Δ*

ΔCompared with control, P < 0.05, *compared with 1 h, P < 0.05

The serum markers of liver injury, such as ALT, AST, STB and LDH, increased with time after ConA injection. Compared with normal control, ALT and STB increased at 1 h by 1.7-fold and 1.8-fold respectively, and ALT, AST, STB and LDH level at 6 h increased by 27.9-fold, 9.0-fold, 2.4-fold and 4.2-fold, respectively.

### Genes screened by Gene-chip study

Genome-wide transcriptional profiling of the liver has demonstrated that extensive gene expression occurs during very early-stage fulminant hepatitis. To investigate the possible gene expression change in our ConA-induced hepatitis model at a very early stage, we performed a gene chip study by using the Affymetrix probe dataset which includes totally 45101 probes. We found when compared with probes in the control group, 1559 probes increased and 1794 probes decreased in the 1 h group, 3040 probes increased and 4160 probes decreased in the 3 h group, and 5466 probes increased and 5445 probes decreased in the 6 h group. Our data indicate that by time after ConA injection more and more gene expression is detectable. The in detail gene expression data are also available at the GEO website under accession number GSE17184: http://www.ncbi.nlm.nih.gov/geo/

### Genes further screened by ANOVA and model profile analysis

ANOVA corrected with the randomized variance model was performed to determine genes that were expressed separately and differentially. As a result, total 1,473 genes that had a *p-value *and the false discovery rate (FDR) less than 0.05 were declared to be significantly expressed. The gene expression value per group was the geometric mean of the Robust Multichip Average (RMA) normalized gene signals of 3 samples per time point.

**Figure 2:**
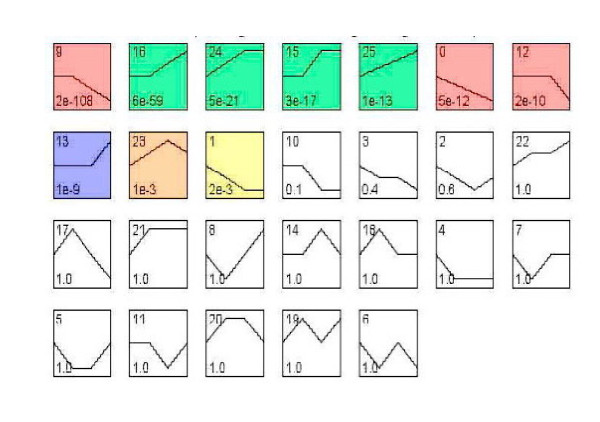
**The expression patterns of 1,473 genes analyzed by model profile**. The expression patterns of 1,473 genes were analyzed and twenty-six model profiles were used to summarize. Each box represents a model expression profile. The upper number in the profile box is the model profile number and the lower one is the *p*-value; Ten expression patterns of genes showed significant *p*-values (*p *< 0.05) (colored boxes)

To further narrow the target genes which harbor great significance among the declared 1,473 genes, we chose to use the twenty-six model profiles to summarize the expression pattern of the genes. As shown in Figure 2, among the 26 patterns, we identified 10 patterns of genes that shown very significant *p-values *(colored boxes).

**Figure 3:**
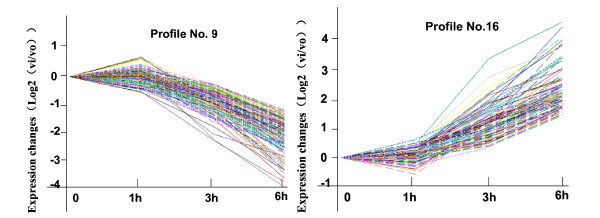
**Gene expression of profile No.9 and No. 16 in the context of significant ConA-induced liver injury**. The profile No.9 (A) contained 209 genes and they decreased in expression consistent with prolonged liver damage after injecting ConA. The profile No.16 (B) had 118 genes and they increased in expression consistent with prolonged liver damage after the injection. These processes may indicate that the assigned genes closely correlate with the results of regulatory gene expression, including activation and inhibition in the context of liver damage caused by ConA challenge. The horizontal axis represents time, and the vertical axis shows the time series of gene expression levels for the gene after Log normalized transformation.

Among these patterns, the two most significant patterns were profiles No. 9 and No.16 according to ascending p-values. While the profile No. 16 contained 118 genes whose expression increased constantly, the profile No. 9 contained 209 genes whose expression reduced constantly, after ConA injection (Figure 3).

### 26 genes identified by Gene Co-expression Network with k-core algorithm from profiles No.9 and No. 16

Genes in profiles No.9 and No.16 were then analyzed and identified by gene co-expression network with k-core algorithm to determine which gene or genes may play pivotal role in the early stage of fulminant hepatitis. Gene networks are constructed from functional gene associations. In the network, cycle nodes represent genes, and edges between two nodes represent interactions between genes, which were quantified by degree. Degrees within the network which describe the number of single gene that regulates other genes represent the size of the cycle node. The higher the degree, the more central the gene occurs within the network. The clustering coefficient can be used to estimate the complexity of interactions among genes that neighbor the core gene with the exception of core gene participation. The lower the clustering coefficient, the more independent of the core gene are the interactions among genes in the neighborhood of the core gene[6].

A k-core of a gene co-expression network usually contains cohesive groups of genes. On the other hand, phylogenetic analysis classifies genes into groups based on the similarity of base sequences[7].

**Figure 4:**
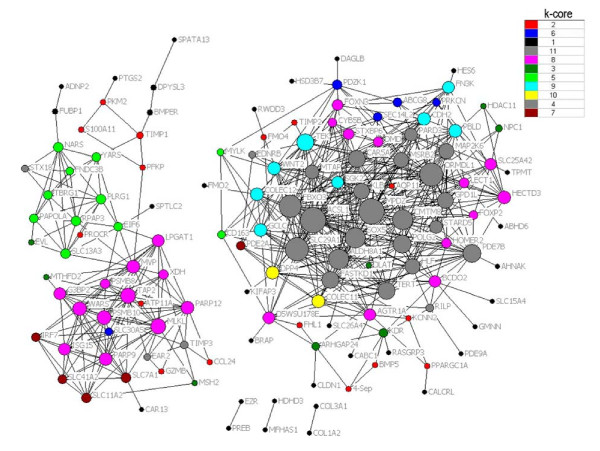
**Gene co-expression network**. Genes from profiles No.9 and No.16 were analyzed and identified by gene co-expression network with k-core algorithm. Cycle nodes represent genes, the size of nodes represents the power of the interrelation among the nodes, and edges between two nodes represent interactions between genes. The more edges of a gene, the more genes connecting to it, the more central role it has within the network.

The k-core subnetwork with higher k-core level in our result is considered to have a core status within a large-scale gene network made up of differential 26 genes (see Figure 4 and Table 2). Most of the genes are attributed to transport, transcription, regulation of transcription, metabolic processes, and carbohydrate metabolic processes, consistent with the gene ontology hierarchical category, lipoprotein catabolic processes, peptide biosynthetic processes, positive regulation of calcium-dependent cell-cell adhesion, fructosamine metabolic processes, male sex differentiation, and regulation of cholesterol biosynthesis. The core genes MPDZ and ACSL1 appear at the center of both the large-scale network and the 11 k-core subnetwork. They directly regulate 34 neighboring genes that interact according to their degrees. These interactions depend in large part on MPDZ and ACSL1 because the clustering coefficients of these genes are 0.38 and 0.42, which are lower than for other genes.

**Table 2:** 26 genes identified by gene co-expression network with k-core algorithm

Gene symbol	Clustering Coefficient	Degree	k-core
ACSL1	0.418894827	34	11

ALDH8A1	0.489230782	26	11

CAR5A	0.589473665	20	11

CMTM8	0.464615375	26	11

FASTKD1	0.594736814	20	11

FBXO3	0.467980295	29	11

GPD1L	0.637426913	19	11

HLF	0.757352948	17	11

KLB	0.608187139	19	11

MAP2K6	0.70588237	17	11

MPDZ	0.377896607	34	11

MSRB2	0.509803951	18	11

MTAP	0.608333349	16	11

ORMDL1	0.430107534	31	11

PARD3	0.619047642	15	11

PDE7B	0.486166	23	11

POLG2	0.565217376	23	11

SLC29A1	0.393548399	31	11

SOX5	0.492307693	26	11

STARD5	0.666666687	15	11

TERT	0.452631593	20	11

COLEC11	0.494505495	14	10

DPP4	0.495238096	15	10

CDH2	0.561904788	15	9

COLEC12	0.604395628	14	9

FN3K	0.551282048	13	9

Degrees describe the number of single gene that regulates other genes represent the size of the cycle node. The higher the degree, the more central the gene occurs within the network. The clustering coefficient can be used to estimate the complexity of interactions among genes that neighbor the core gene with the exception of core gene participation. The lower the clustering coefficients, the more independent of the core gene are the interactions among genes in the neighborhood of the core gene. A k-core of a gene co-expression network usually contains cohesive groups of genes. The higher the k-core, the more central the genes occurs within the network. In this study, the top k-value is 11, which is considered to have a core status within a large-scale gene network made up of differential genes from profiles No.9 and No. 16.

In this network, transmembrane proteins, such as MPDZ and Acsl1, were shown to play a role in the important processes of cell adhesion, carbohydrate metabolism and molecular transport. However, MPDZ expression constantly decreased over the four time points. It may contribute to damage to cell junctions in liver cells. The result of MPDZ down expression may be related to disorders of liver tissue and may lead to obstacles in the carbohydrate metabolic process. Acs11 was an essential gene that was associated with long-chain-fatty-acid-CoA synthase activity. The under expression of the gene gives rise to a series of obstructions in lipid and cholesterol biosynthetic and metabolic processes.

### Real-time RT-PCR verification of the genes

Real-time RT-PCR was used to confirm the expression of the 26 genes. Compared with normal controls, the expression of most of the 26 genes showed a statistical significant change (*p < 0.05*). Expressions of PARD3, ALDH8A1, CMTM8 and FBXO3 were also higher than those of normal controls, but there was no statistical significance (*p > 0.05*) (See details in Table 3 and the primers see in Additional file 1).

**Table 3:** The expression of 26 genes in the very early stage of fulminant hepatitits mice model by real-time RT-PCR

genes	Number	ΔΔct (1 h/control)	ΔΔct (3 h/control)	ΔΔct (6 h/control)	ΔΔct (12 h/control)
ACSL1	12	1.58 ± 1.72	2.21 ± 3.81	0.47 ± 0.55#	0.71 ± 1.46

ALDH8A1	12	1.26 ± 0.62	5.16 ± 9.96	16.47 ± 40.57	2.78 ± 3.74

CMTM8	12	4.24 ± 5.47	60.46 ± 127.18	48.60 ± 111.88	62.93 ± 106.90

COLEC11	12	0.73 ± 0.29#	4.04 ± 2.35#*	3.27 ± 4.40	1.50 ± 2.00&

COLEC12	12	1.19 ± 0.54	3.86 ± 3.41#*	2.44 ± 2.77	6.59 ± 12.03

DPP4	12	0.87 ± 0.60	11.64 ± 6.76#*	14.91 ± 14.25#*	86.16 ± 218.29

FBXO3	12	1.79 ± 1.59	2.13 ± 1.91	1.64 ± 1.54	1.09 ± 0.72

FN3K	12	1.60 ± 1.98	3.38 ± 2.88#	2.01 ± 1.96	2.20 ± 3.57

GPD1L	12	2.61 ± 3.59	0.76 ± 0.83#	0.35 ± 0.30#	1.25 ± 3.02

HLF	12	1.49 ± 1.39	8.15 ± 10.11*#	4.26 ± 5.27	3.72 ± 5.31

KLB	12	1.01 ± 0.51	1.93 ± 1.11*#	2.93 ± 5.05	1.58 ± 1.91

MAP2K6	12	2.61 ± 1.75#	31.29 ± 90.44	0.94 ± 1.188*	1.40 ± 2.07

MPDZ	12	3.02 ± 2.23#	6.28 ± 4.92#	3.96 ± 5.98	5.11 ± 6.11

MTAP	12	1.77 ± 0.74#	11.75 ± 31.48	3.81 ± 8.55	1.56 ± 1.56

MSRB2	12	1.77 ± 1.60	7.07 ± 5.81#*	2.38 ± 2.35&	3.34 ± 3.52#

ORMDL1	12	1.15 ± 0.91	2.85 ± 1.81#*	1.57 ± 0.86&	1.23 ± 0.50&

POLG2	12	0.71 ± 0.51	5.74 ± 9.951	3.07 ± 3.28*	1.46 ± 0.84*

STARD5	12	1.39 ± 1.20	2.58 ± 5.52	2.09 ± 2.91	2.52 ± 1.03#*

PARD3	12	1.24 ± 1.50	6.74 ± 11.47	3.83 ± 4.79	2.05 ± 2.39

SLC29A1	12	0.82 ± 0.44	1.75 ± 1.35*	1.16 ± 1.71	0.33 ± 0.29#*&

SOX5	12	0.91 ± 0.65	1.56 ± 0.63#*	0.85 ± 0.52&	3.25 ± 3.84

TERT	12	0.82 ± 0.70	2.71 ± 2.52#*	1.89 ± 2.55	1.09 ± 2.22

CAR5A	12	1.87 ± 2.61	2.56 ± 3.16	0.54 ± 0.57#	0.45 ± 0.63#&

FASTKD1	12	0.38 ± 0.26#	1.75 ± 1.35*	0.95 ± 0.50*	0.46 ± 0.30#&◇

PDE7B	12	0.64 ± 0.40#	1.39 ± 2.18	0.44 ± 0.33#	0.59 ± 0.33#

FASTKD1	12	0.96 ± 0.96	1.90 ± 1.04#*	0.63 ± 0.44#&	0.60 ± 0.49#&

#: Compared with 0 h, the difference was significant (p < 0.05)

*: Compared with 1 h, the difference was significant (p < 0.05)

&: Compared with 3 h, the difference was significant (p < 0.05)

◇: Compared with 6 h, the difference was significant (p < 0.05)

Compared with normal controls, the expression of most of the 26 genes showed a statistical significant change (p < 0.05). Expressions of PARD3, ALDH8A1, CMTM8 and FBXO3 were also higher than those of normal controls, but there was no statistical significance (p > 0.05)

## Discussion

In this study, use of a fulminant hepatitis mouse model, we have focused on hepatic gene expression during early stages of fulminant hepatitis. By a series of biological and bioinformatics analysis, we identified 26 genes whose expression are significantly altered at the very early stage and have shown big correlations with fulminant hepatitis.

Using gene chips has the advantage of generating huge amounts of information. The high cost of microarray experiments and limited sample availability resulted in a small sample size for each treatment group. In addition, because we had to filter differential genes, our two-group comparison accuracy was reduced because variance estimates have few degrees of freedom. Consequently, we obtained results with more power and less variation by using the random variance model corrected standard t-test or ANOVA methodology [8].

We predefined certain sets of profiles in the context of patterns that can be expected in the course of growth. On account of the noise and the small number of points for each gene, any one pattern will be shared by many genes and can be expected to appear by chance. First, we selected a set of potential expression profiles. These sets of profiles cover all of the possible profiles that can be generated by gene expression in the course of growth, and each represents a single temporal expression pattern. Enrichment of genes in each profile was then used to measure the profile significance level. Significant profiles may indicate that there were mostly common functions attributed to genes that are co-expressed. Such functions mainly influence the biological character [9].

In biological processes, macromolecular networks can be constructed by the experience results from such as Y-2H, and coimmunoprecipitation, or from the algorithmic predict base on the gene function correlation and expression profiles. Because of the flexibility of the network model base on the algorithmic predict from high throughput gene expression tests, we can look at snapshots of protein-protein interaction, gene expression regulatory networks and metabolism networks among different groups. The intrinsic gene networks of a phenotype can represent gene function propriety of sample. k-cores and degrees of genes are key attributes in the network. A k-core of a network is a subgraph in which all genes are connected to at least k other genes in the subgraph [6]. In a protein-protein interaction network, a node represents a protein and an edge represents an interaction between proteins. In this work, we use k-cores of protein-protein interaction networks to define main gene function in main subgraph. The rank of k-core value describes the complexity of gene association relationship. The complexity of gene relationship increased with k-core value rank. We wanted to find main Gene Ontology (GO) assigned by the maximum numbers of genes in separately k-core and then define the key gene functions at each complexity level of network [7]. For this analysis result, we conclude the core functions at the core status of network which have a top k-core level.

Regulation of gene expression as well as protein-protein interactions can change in a dynamic process over time. Such processes may exhibit a regulatory character. These macromolecular networks respond to the events such as growth, metabolism, and apoptosis. Because certain gene interactions are only observable under very specific conditions, we combined experimental data to model co-expression networks from different types of experiments. One of the important reasons for doing this was the fact that differential macromolecular network structures have different sources. In this study, twenty-six genes were selected using the network, and validated by Real-time RT-PCR. Compared with normal controls, the expression of most of the 26 genes appeared a statistical significant change (p < 0.05).

A very important issue about the results from a gene-chip-based analysis is their correlation to the actual biological processes. Many of the 26 genes identified in this study including MPDZ and ACSL1 are involved in the metabolism of liver cells. Although the expression of these genes may not be specifically initiated or suppressed by fulminant hepatitis, the emergence or inhibition of the genes at the very early stage of fulminant hepatitis could be followed by severe liver damage, the pathological hallmark of fulminant hepatitis presented as large area of necrosis, thereby supplying potential diagnostic information for clinical fulminant hepatitis. In this study, we employed a ConA-induced animal fulminant hepatitis model instead of the use of human tissue which is extremely tough to obtain. As mentioned earlier, ConA-induced hepatitis has broadly been used as an experimental model of immune-mediated liver disease. Our pathological and biochemical data further confirmed that the model resembles very much the human fulminant hepatitis. Therefore, our results may provide important referential merit for clinical investigation. Nevertheless, in all conscience, the genes identified here are required to be further dissected and confirmed in human by other clinic-related studies.

## Conclusions

At the early stages of fulminant hepatitis induced by ConA, expression of twenty-six genes involved in protein transport, transcription regulation and cell metabolism altered significantly. These genes form a network and have shown strong correlation with fulminant hepatitis development and a promising future for early diagnosis. Our study provides several potential targets for the early diagnosis of fulminant hepatitis in clinical scenario.

## Methods

### Animals

Eight- to 10-week-old male Balb/C mice were purchased from the Animal Center, Zhejiang Academy of Medical Sciences. The average weight was 25 g. All mice were kept at least 1 wk at 22°C and 55% relative humidity in a 12-h day/night lighting environment with free access to food and water. The animals were raised and cared for in accordance with guidelines established by the National Science Council of the Republic of China.

### ConA administration

Mice were given a tail vein injection of ConA (Sigma Chemical Co., St. Louis, MO) in 100 μl PBS at a concentration of 20 mg/kg, and same dose of PBS was used as negative control.

### Histology

The livers were removed, and part of each liver was frozen in liquid nitrogen, while another part was fixed with 4% phosphate-buffered paraformaldehyde and embedded in paraffin. Tissue sections (4 μm) were prepared and stained with hematoxylin/eosin; sections were then examined under light microscopy.

### Biochemical detection

The extent of liver injury was assessed by determining serum ALT, AST, STB and LDH activity levels using the standard Reitman-Frankel method.

### Isolation of RNA

Liver samples from very early stage of fulminant hepatitis mouse model, which include control group, 1 hour 3 hours and 6 hours after the injection of ConA, were ground into a fine powder in a mortar cooled by liquid nitrogen, and 100 mg was added to 1 ml prechilled Trizol reagent (Invitrogen, Carlsbad, CA). Total RNA extractions were performed according to the manufacturer's directions, and the RNA was further purified by passage through RNeasy mini-columns (QIAGEN, Valencia, CA) according to the manufacturer's protocols for RNA clean-up. Final RNA preparations were resuspended in RNase-free water and stored at -80°C. The RNA samples were quantified spectrophotometrically, and purity and integrity were assessed by agarose gel electrophoresis. All samples exhibited 260/280 absorbance ratios of approximately 2.0, and all showed intact ribosomal 28S and 18S RNA bands in an approximate ratio of 2:1 as visualized by ethidium bromide staining.

### First-Strand cDNA Synthesis

Total RNA (1 μg to 15 μg) and 2 μL of 50 μM T7-Oligo (dT) Primer were mixed together in a 0.2 mL PCR tube, and RNase-free water was added to create a final volume of 12 μL. The tubes were gently flicked a few times to mix the solution, and then the tubes were briefly centrifuged to collect the reaction products and reagents at the bottom of the tube. The reaction was incubated for 10 minutes at 70°C. The sample was cooled at 4°C for at least 2 minutes. Subsequently, 4 μL of 5× 1st Strand Reaction Mix, 2 μL of 0.1 M DTT, 1 μL of 10 mM dNTP, were combined and mixed thoroughly by flicking the tube a few times. The tubes were centrifuged briefly to collect the reaction products and reagents at the bottom of the tube, and the tubes were immediately placed in a 42°C environment. SuperScript II (1 μL) was added to each RNA sample for a final volume of 20 μL. The RNA was incubated for 1 hour at 42°C; finally, the sample was cooled for at least 2 minutes at 4°C.

### Second-Strand cDNA Synthesis

RNase-free water 91 μL, 30 μL of 5× 2nd Strand Reaction Mix, 3 μL of 10 mM dNTP, 1 μL of *E. coli *DNA ligase, 4 μL of *E. coli *DNA Polymerase I, and 1 μL of RNase H were mixed by gently flicking the tube a few times. The tubes were centrifuged briefly to collect solids at the bottom of the tube. We added 20 μL of the First-Strand cDNA synthesized as described above. The tube was flicked gently a few times to mix the solution, and then the samples were centrifuged briefly to collect any reaction products and reagents at the bottom of the tube. The cDNA was incubated for 2 hours at 16°C. 2 μL of T4 DNA Polymerase was added to each sample, and the tubes were incubated for 5 minutes at 16°C. Finally, we added 10 μL of 0.5 M EDTA.

### Microarrays

The biotinylated cDNAs were hybridized to 12 individual Affymetrix GeneChips Mouse 430 2.0 Arrays (Affymetrix, Inc., Santa Clara, CA). This product analyzes the expression level for over 39,000 transcripts and variants, including over 34,000 well-substantiated mouse genes.

### Data Analysis

#### Significant Differential Gene Analysis

The per-group gene expression value was the geometric mean of RMA normalized gene signals from 3 samples per time point. Due to the fact that the number of samples per class (i.e., three samples per group) was far lower than the number of genes, there were few degrees of freedom for the gene expression signal variance. The low number of degrees of freedom makes an accurate estimation of variability difficult. Significant differential genes among the three groups were filtered by ANOVA and corrected by the random variance model (RVM)[8]. In this experiment, the differential genes from different time points were filtered by RVM ANOVA. To assess the significance of a particular sample having occurred by random chance, the false discovery rate (FDR) was estimated to allow us to determine whether certain genes were actually significant by repeating the comparison test and the permutation test 1000 times. The threshold of truly significant genes was taken to be *p-value *< 0.05 and *FDR *< 0.05.

#### Time Sequence Profile Analysis of Gene Expression

We first select a set of distinct and representative temporal expression profiles independent of the data. These model profiles correspond to possible profiles of a gene's change in expression over time. Passing the data normalization (, , , and ), each gene is assigned to the model profile that most closely matches the gene's expression profile as determined by the correlation coefficient. Since the model profiles were selected independent of the data, the algorithm can then determine which profiles have a statistically significant higher number if genes assigned using a permutation test. This test determines an assignment of genes to model profiles using a large number of permutations of the time points. It then uses standard hypothesis testing to determine which model profiles have significantly more genes assigned under the true ordering of time points compared to the average number assigned to the model profile in the permutation runs. Significant model profiles can either be analyzed independently, or grouped together based on similarity to form clusters of significant profiles.

#### Construction and Topological Analysis of the Gene Co-Expression Network

In biological processes, macromolecular networks can be constructed by using results from experiments involving Y-2H, coimmunoprecipitation [10], or algorithmic predictions that may be based on gene function correlation and expression profiles [11]. Because of the flexibility of network models based on algorithmic predictions that derive from high-throughput gene expression tests, we can look at snapshots of protein-protein interactions, gene expression regulatory networks and metabolic networks among different groups [12]. Gene networks are constructed from functional gene associations. In the network, cycle nodes represent genes, and edges between two nodes represent interactions between genes. As network elements represent the ways in which genes may regulate other genes, large scale gene networks can be divided into certain subgraphs, named k-core networks, in which all genes are connected to at least k other genes in the subgraph [7]. As a result, the rank of k-core value describes the complexity of the gene association relationship. Cycles with identical colors are part of the same subgraph. In light of the definition of k-core networks, core status within large scale gene networks consists of subgraphs that are associated with higher k values. The complexity of gene relationships increases with k-core value rank. The intrinsic gene network of a given phenotype can represent gene function propriety within a sample. In this work, we use k-cores of protein-protein interaction networks to define major gene functions in the main subgraph. We wanted to identify the main GO on the basis of the maximum number of genes in separate k-cores and then we wished to define the key gene functions at each complexity level of the network [13]. We transformed the normalized expression value of the Pearson correlation into measures of pairwise connection strengths [12]. We selected genes from the two most significant profiles, namely profiles 9 and 16, to construct a co-expression network. The network edges were specified to feature correlation coefficients of above 0.9, to ensure strong gene co-expression relationships. The centrality of the network is represented by the central degree [6]. It is possible to identify characteristic distance variables among genes. We attempted to describe the contribution that gene i makes to the status (i.e., the determining central status) of gene j in the network. The maximum core order is termed the main core or the highest k-core of the graph[7]. A k-core subgraph can be generated by recursively deleting the vertices from a graph whose degree is less than k [7]. Larger values clearly correspond to vertices with larger degrees and more central positions in the network structure. In this work, we apply the notion of k-core subgraphing to predict gene function similarity [13]. The nodes labeled by the same color may represent similar gene ontology terms. The highlighted ontologies of same-colored nodes were assessed by counting genes that featured the same gene ontology and the same color.

#### Real-time RT-PCR confirmation of differentially expressed genes

Real-time RT-PCR was used to verify the differential expression of twenty-six genes that were detected by the Affymetrix GeneChip. The primers used are listed in Additional file 1. Each real-time RT-PCR reaction (in 25 μL) contained 2×SYBR Green Realtime PCR Master Mix, 0.4 μM primers, and 0.5 μL of template cDNA. The cycling conditions consisted of an initial, single cycle of 5 min at 95°C, followed by 40 cycles of 30 sec at 95°C, 30 sec at 54°C, 15 sec at 72°C, and fluorescence acquisition at 83°C for 1 sec. The cDNA was synthesized using reverse transcriptase (Promega), oligo(dT) and random primers with 5 μg RNA from the same samples as those used in the microarray. The PCR amplifications were performed in duplicate for each sample. The gene expression levels were quantified relative to the expression of β-actin by employing an optimized comparative Ct (ΔΔCt) value method. The differences in gene expression levels between groups were compared using the Student's t-test. A *p value *< 0.05 was considered significant.

## Authors' contributions

FC and ZC conceived of the study and participated in its design and coordination. HHZ and LFZ performed the data analysis, LYZ and JW performed the experiment of animal model, FC and WL drafted the manuscript, and JL and SSW performed RT-PCR. All authors contributed to and approved the final manuscript.

## Supplementary Material

Additional file 1 **Primers of 26 genes selected from gene co-expression network**. In this table list 26 upsream primers and downstream primers used for RT-PCR. Click here for file
