# Supplementary material for: Genes related to the very early stage of ConA-induced fulminant hepatitis: a gene-chip-based study in a mouse model
Source: BMC Genomics. 2010 Apr 15;11:240. doi: 10.1186/1471-2164-11-240 (PMC2867829; doi:10.1186/1471-2164-11-240)
Supplement: Additional file 1 — Primers of 26 genes selected from gene co-expression network. In this table list 26 upsream primers and downstream primers used for RT-PCR. [file 1471-2164-11-240-S1.DOC]

**Additional files**

Additional file 1 Primers of 26 genes selected from gene co-expression network

| acsl1 | Upstream primer | CGACTTGTTGAAACTTGG |
| --- | --- | --- |
|  | Downstream primer | TCATCTATCTGCGACCTG |
| Aldh8a1 | Upstream primer | GATGACTCAGTGGGTAGCG |
|  | Downstream primer | TTGATGGTGCGTTGTTTCT |
| Car5a(Human：CA5A) | Upstream primer | CAGGTGGAGTTTGACGATT |
|  | Downstream primer | CCACGGAGGCTTTCTTGTA |
| Cdh2 | Upstream primer | CCCAAGTCCAACATTTCC |
|  | Downstream primer | CCGCCGTTTCATCCATA |
| Cmtm8 | Upstream primer | TCTTTGCCTTCCTGGTCA |
|  | Downstream primer | AGTGGGATTCGTTCTGGT |
| Colec11 | Upstream primer | CTCAGGTGGCTGGAATGATG |
|  | Downstream primer | TGCTCAGGGTTAGTTTGTG |
| colec12 | Upstream primer | TCAGAACAGGAAAGCGAATG |
|  | Downstream primer | GCGTAAATCAAGCCAGCA |
| Dpp4 | Upstream primer | CCACGCAAGGGAGCAAC |
|  | Downstream primer | TAGGCGAGCCAGGAAGTA |
| fastkd1 | Upstream primer | GGGAACTGAATGACTTGGTA |
|  | Downstream primer | AGCAGCAGAGTGTTGAGGTA |
| fbxo3 | Upstream primer | GTTGTATCAGGTGGTTTCC |
|  | Downstream primer | TTGTTATCCTCCAGTAGCG |
| fn3k | Upstream primer | CTTGGTGCGGGTTCCTA |
|  | Downstream primer | CTTTGATGCCTGACTGCT |
| Gpd1l | Upstream primer | CCGTCAAGATGTGGGTC |
|  | Downstream primer | TGGATGAACTGGTGAGGGAT |
| Hlf | Upstream primer | GTGACGACTCCTGCTCCA |
|  | Downstream primer | CCATACTCCATTCCAACGA |
| Klb | Upstream primer | CAACCGCACGAGTAATGA |
|  | Downstream primer | CCCAGTCGCAATGTAAGGA |
| map2k6 | Upstream primer | TTTGAACAGCCTCAGACC |
|  | Downstream primer | ACGCATCTTCTCCACCAC |
| Mpdz | Upstream primer | AGCCTACCTGTGACGAACT |
|  | Downstream primer | AACAACACTAAAGCCGAG |
| Mtap | Upstream primer | GGCGGTGAAGATTGGAA |
|  | Downstream primer | ATGTTTGCCTGGTAGTTGA |
| msrb2 | Upstream primer | AGCAAGCCCTGACTGTTT |
|  | Downstream primer | GTCCCACCCTGTGGTCTC |
| Ormdl1 | Upstream primer | AATGAACAGCCGTGGTA |
|  | Downstream primer | TTCCGAGAAGATGTAAACT |
| Pde7b | Upstream primer | ATGCTAAGCCATCTCGC |
|  | Downstream primer | GTCTGCTCCAGGGTCTC |
| Polg2 | Upstream primer | AACAGCAATCAGACACCCAG |
|  | Downstream primer | AGAAGCCGATGACGCAAC |
| Stard5 | Upstream primer | CGGGAGAAGTGGGATGA |
|  | Downstream primer | CCCTGGGAGAAATAAGC |
| Pard3 | Upstream primer | GCAGCAAACAAGGAGCAAT |
|  | Downstream primer | TGAGGCGTGAGCACTATG |
| Slc29a1 | Upstream primer | GCCGAGAACACTGGAGCC |
|  | Downstream primer | CTTGGTTGCGGTCATAAA |
| Sox5 | Upstream primer | TGATGATTCCCGTGTTCC |
|  | Downstream primer | TTGTGCTCTTGTCTGTGTGA |
| tert | Upstream primer | TTGAAGTGTCACGGTCTATT |
|  | Downstream primer | CACGCTGGTCAAAGGGA |

In this table list 26 upsream primers and downstream primers used for RT-PCR.
